# Association between nocturnal activity of the sympathetic nervous system and cognitive dysfunction in obstructive sleep apnoea

**DOI:** 10.1038/s41598-021-91329-6

**Published:** 2021-06-07

**Authors:** Ridwan M. Alomri, Gerard A. Kennedy, Siraj Omar Wali, Faris Alhejaili, Stephen R. Robinson

**Affiliations:** 1 Department of Psychology, College of Social Sciences, University of Jeddah, Jeddah, Saudi Arabia; 2 School of Health and Biomedical Sciences, RMIT University, Bundoora, VIC Australia; 3 Institute for Breathing and Sleep, Austin Health, Heidelberg, VIC Australia; 4 Sleep Medicine and Research Centre, King Abdulaziz University Hospital, Jeddah, Saudi Arabia; 5 School of Science, Psychology and Sport, Federation University, Ballarat, VIC Australia

**Keywords:** Neuroscience, Physiology, Biomarkers, Medical research

## Abstract

Obstructive sleep apnoea (OSA) is associated with repetitive breathing obstructions during sleep. These episodes of hypoxia and associated arousals from sleep induce physiological stress and nocturnal over-activation of the sympathetic nervous system (SNS). One consequence of OSA is impairment in a range of cognitive domains. Previous research into cognitive impairment in OSA have focussed on intermittent hypoxia and disrupted sleep, but not nocturnal over-activation of the SNS. Therefore, we investigated whether nocturnal over-activity of the SNS was associated with cognitive impairments in OSA. The extent of nocturnal SNS activation was estimated from heart rate variability (HRV), pulse wave amplitude (PWA) and stress response biomarkers (cortisol and glucose levels). OSA severity was significantly associated with PWA indices and the HRV low frequency/ high frequency ratio (*p* < 0.05). Morning blood glucose levels were significantly associated with the duration of a blood oxygen saturation (SaO_2_) < 90% (p < 0.01). PWA and HRV were significantly associated with the time taken to perform a task involving visuospatial functioning (p < 0.05), but not with impairments in sustained attention, reaction time or autobiographical memory. These results suggest that the visuospatial dysfunction observed in people with OSA is associated with increased nocturnal activity of the SNS.

## Introduction

Obstructive sleep apnoea (OSA) is a sleep-related breathing disorder that involves repetitive obstruction of breathing during sleep, which is caused by the narrowing and/or complete collapse of the upper airway. One consequence of OSA is the impairment of a wide range of cognitive domains including memory, attention, psychomotor speed and visuospatial skills^1^, and studies have shown that hypoxia and sleep fragmentation contribute to these impairments^2–5^. However, as these two factors only account for 30% to 40% of the variance^6^, additional factors must be involved. Studies of healthy, middle-aged subjects have shown that SNS over-activity during wakefulness is associated with cognitive impairment^7^. The episodes of hypoxia and/or arousals that accompany breathing cessation provoke a stress response, which is indicated by increased activation of the sympathetic nervous system (SNS)^8^. In spite of that, severe hypoxia has been documented to be associated with higher SNS activity^9^. Therefore, one potential mechanism behind the cognitive impairments seen in OSA may be nocturnal overactivation of the SNS.

When healthy individuals fall asleep, the level of SNS activation decreases and heart rate and blood pressure are reduced accordingly^10^. In OSA patients, the activation of the SNS is increased as a function of the duration and severity of apnoeic events rather than by the stages of sleep^11^. Heart rate variability (HRV), a non-invasive method that is based on the differences in the time interval between individual heartbeats, is used to evaluate autonomic nervous system modulation^12^. Sequeira, et al. ^13^ reviewed HRV in adult OSA patients and concluded that people with OSA exhibit reduced vagal tone and higher sympathetic sensitivity. Decreases in pulse wave amplitude (PWA) is another indicator of increased SNS activation^14^. The PWA signal is measured via plethysmography, which is linked to blood flow in the fingers^15^. PWA drops are used to detect vasoconstriction, which reflect autonomic activation^16,17^. Randerath et al.^18^ found that untreated OSA patients displayed more PWA drops during sleep than OSA patients who received treatment with continuous positive airway pressure (CPAP) therapy. Serum cortisol and glucose levels also increase as a result of the stress response during sleep in OSA patients^19,20^; however, adherence to CPAP treatment has been shown to decrease the levels of these stress biomarkers^21,22^.

In this study, we examined whether nocturnal over-activation of the SNS was associated with impairments in the domains of sustained attention, reaction time, autobiographical memory and visuospatial skills, and whether SNS over-activation was correlated with OSA severity. We hypothesised that SNS over-activation would be associated with cognitive impairments in OSA.

## Results

Seventy-two participants met the study inclusion criteria (48 males and 26 females) with a mean age of 40.1 years (standard deviation (SD) of 12.8 years) and a mean body-mass index (BMI) of 33.3 (SD = 9.1 kg/m^2^). Polysomnography results revealed that 12 of the 74 participants did not have OSA, while 26, 16 and 20 participants had mild, moderate and severe OSA, respectively.

A comparison of the demographic characteristics, daytime sleepiness, nocturnal SNS indices and polysomnography data between the four OSA severity groups is shown in Table 1. The severe and moderate groups were significantly older than those in the non-OSA group. There was no variation in BMI, depression or Epworth Sleepiness Scores (ESS) among the groups. From the polysomnography, we found that the duration of SaO_2_ below 90% and the respiratory arousal index were significantly increased with OSA severity [based on the Apnoea-hypopnoea index (AHI)]. No significant differences were found between the OSA severity groups in terms of sleep efficiency, sleep onset latency time, or the durations of non-rapid eye movement (NREM) sleep stages 2 and 3 (i.e. the percentage of the sleep-period time). However, the duration of NREM sleep stage 1 sleep was significantly longer in the severe OSA group, but the rapid eye movement (REM) was significantly shorter in the severe OSA group. For the nocturnal SNS indices, both the PWA drop index and PWA drop duration index increased significantly with OSA severity. In addition, the HRV low frequency/high frequency (LF/HF) ratio was significantly higher in the severe OSA group than in the non-OSA group. However, there were no significant differences in the morning urine and blood cortisol levels and blood glucose levels between the OSA severity groups.

**Table 1:** Comparison of demographic variables, depression, daytime sleepiness, polysomnography parameters and nocturnal sympathetic nervous system indices by OSA severity group.

Variable	Non-OSA (n = 12)	Mild OSA (n = 26)	Moderate OSA (n = 16)	Severe OSA (n = 20)	p-value
	Mean (SD)	Mean (SD)	Mean (SD)	Mean (SD)	
Age (years)	33.7 (14.8)^3,4^	37.4 (11.5)	44.1 (12.4)^1^	46.5 (10.4)^1^	**< 0.01**
Body Mass Index (kg/m^2^)	30.2 (9.7)	31.2 (8.1)	33.3 (9.9)	37.3 (9.9)	0.09
Current smoker (%)^+^	1 (8)	5 (22)	4 (14)	7 (42)	0.13
DASS-21 depression subscale	8.3 (8.3)	13.0 (9.3)	11.5 (10.7)	12.2 (8.8)	0.51
ESS	9.1 (5.3)	9.0 (3.9)	11.0 (6.7)	13.3 (6.4)	0.06
Apnoea-hypopnea Index (AHI)	2.3 (1.3)^3,4^	9.2 (2.9)^3,4^	21.1 (4.9)^1,2,4^	49.2 (20.6)^1,2,3^	**< 0.01**
Oxygen Desaturation Index (ODI)	2.6 (1.8)^4^	9.5 (6.9)^4^	16.8 (13.7)^4^	47.9 (25.9)^1,2,3^	**< 0.01**
SaO_2_ time < 90% (mins)	0 (1)^4^	2 (7)^4^	11 (28) ^4^	32 (48)^1,2,3^	**< 0.01**
Minimum SaO_2_	90.0 (3.9)^4^	88.6 (3.32)^4^	85.1 (7.2)^4^	78.2 (10.0)^1,2,3^	**< 0.01**
Total sleep time (mins)	299 (64)^2,4^	248 (65)^1^	270 (46)	230 (60)^1^	**0.01**
Sleep efficiency (%)	78.1(13.4)^2,4^	63.7 (14.8)^1^	73.3 (10.8)	63.2 (15.5)^1^	**< 0.01**
Sleep latency time (mins)	19 (14)	50 (48)	30 (21)	35 (28)	0.07
Waking after sleep onset time (mins)	63 (51)	73 (48)	68 (38)	99 (47)	0.08
Respiratory Arousal Index	1.4 (1.1)^3,4^	5.5 (4.9)^4^	11.2 (4.9)^1,4^	26.9 (16.9)^1,2,3^	**< 0.01**
N1 time duration (%)	08 (04)^4^	11 (8)^4^	12 (7) ^4^	22 (13)^1,2,3^	**< 0.01**
N2 time duration (%)	49 (10)	45 (12)	55 (16)	40 (13)	0.23
N3 time duration (%)	22 (09)	26 (13)	21 (16)	119 (20)	0.53
REM time duration (%)	14 (08)^3,4^	09 (07)	07 (06)^4^	06 (07)^4^	**0.01**
PWA drop index	2.7 (3.0)^4^	3.4 (5.5)^4^	6.0 (4.6)^4^	12.3 (7.0)^1,2,3^	**< 0.01**
PWA drop time duration index	2.1 (2.6) ^4^	2.9 (2.6)^4^	4.0 (3.2)^4^	9.6 (6.0)^1,2,3^	**< 0.01**
HRV LF/HF ratio (log)	0.1 (0.6)^4^	0.04 (0.8)	0.04 (0.8)	0.6 (0.7)^1^	**0.04**
Morning urine cortisol (mmol/L)	75.1 (67.5)	90.2 (143.0)	102.5 (177.3)	152.0 (163.4)	0.20
Morning blood cortisol (mmol/L)	232.0 (115.2)	240.3 (138.0)	292.0 (135.8)	287.0 (122.0)	0.13
Morning blood glucose (mmol/L)	5.2 (0.9)	6.0 (1.9)	5.4 (1.0)	6.0 (2.3)	0.39

OSA severity cut-off values were: 5–14 AHI (mild); 15–29 AHI (moderate); ≥ 30 AHI (severe); OSA, obstructive sleep apnoea; Significant differences between groups were defined by: ^1^p < 0.05 vs. non-OSA; ^2^p < 0.05 vs. mild OSA; ^3^p < 0.05 vs. moderate OSA; ^4^p < 0.05 vs. severe OSA; + : Pearson’s chi-square; *Log* logarithmic transformation, *DASS-21* depression, anxiety and stress scale-21, *ESS* Epworth sleepiness scale, *SaO*_*2*_ arterial blood oxygen saturation, *PWA* pulse wave amplitude, *HRV* heart rate variability, *LF* low frequency, *HF* high frequency, *N1* sleep stage 1, *N2* sleep stage 2, *N3* sleep stage 3, *REM* rapid eye movement sleep stage.

Bolded p-values indicate statistically significant effects

In addition, there were no significant differences in the cognitive test scores between the OSA groups, except for total episodic memory and episodic childhood memory, which were positively correlated with OSA severity (Table 2).

**Table 2:** Comparison of cognitive tests by OSA severity group.

Variable	Non-OSA (n = 12)	Mild OSA (n = 26)	Moderate OSA (n = 16)	Severe OSA (n = 20)	p-value
	Mean (SD)	Mean (SD)	Mean (SD)	Mean (SD)	
PVT RT	317.1 (73.5)	296.0 (43.3)	323.8 (72.4)	323.8 (72.5)	0.53
PVT RT slowest 10%	569.5 (204.9)	478.5 (104.7)	591.9 (310.6)	549.9 (223.2)	0.34
PVT RT-10-lapses > 500 ms	5.2 (9.8)	3.0 (3.4)	4.3 (3.4)	5.1 (9.5)	0.78
AM-time (mins)	35.9 (20.0)	38.5 (12.4)	45.5 (11.9)	42.7 (18.3)	0.433
AM-errors	10.0 (6.6)	14.7 (8.8)	12.2 (5.6)	10.7 (6.5)	0.16
Total semantic memory	58.6 (3.7)	56.8 (4.9)	57.8 (4.3)	58.1 (3.8)	0.26
Childhood semantic memory	19.5 (1.7)	18.0 (3.1)	19.4 (1.6)	19.5 (1.9)	0.11
Early adult life semantic memory	19.0 (2.1)	19.0 (2.7)	19.2 (2.0)	19.2 (2.4)	0.98
Recent life semantic memory	20.0 (1.6)	18.4 (2.6)	19.6 (1.7)	19.5 (1.7)	0.09
Total episodic memory	22.1 (4.5)^2,4^	16.8 (5.9)^1,4^	17.2 (5.1)	15.8 (5.4)^1,2^	**0.01**
Episodic childhood memory	7.8 (1.6)^2,4^	5.6 (2.6)^1,4^	5.8 (2.1)	5.1 (2.3)^1,2^	**0.01**
Episodic early adult life memory	6.8 (2.2)	5.3 (2.4)	5.9 (1.9)	5.1 (2.5)	0.17
Episodic recent life memory	7.5 (1.5)	5.8 (2.7)	5.6 (2.1)	5.5 (2.1)	0.05

OSA severity cut-off values were: 5–14 AHI (mild); 15–29 AHI (moderate); ≥ 30 AHI (severe); Significant differences between groups were defined by: ^1^p < 0.05 vs. non-OSA; ^2^p < 0.05 vs. mild OSA; ^3^p < 0.05 vs. moderate OSA; ^4^p < 0.05 vs. severe OSA; PVT, psychomotor vigilance test; RT, reaction time; AM, Austin Maze; ms, milliseconds.

Bolded p-values indicate statistically significant effects

Multiple linear regression analyses (Table 3) demonstrated that nocturnal PWA was significantly associated with the respiratory arousal index. In addition, the HRV LF/HF ratio was associated with age and the respiratory arousal index. Only the morning blood glucose level was significantly associated with an SaO_2_ time < 90%. Urine and blood cortisol levels were not significantly associated with any of the OSA predictors.

**Table 3:** Multiple regression analysis for age, SaO_2_ time < 90%, respiratory arousal index and body mass index to predict nocturnal pulse wave amplitude, heart rate variability and morning blood glucose levels.

Variable	R^2^	p_model_	Predictor	B	SE	β	sr	p-value
PWA drop index	0.40	< 0.01*	Body Mass Index	0.10	0.08	0.14	0.13	0.19
			Age (years)	0.08	0.06	0.14	0.13	0.19
			Respiratory Arousal Index	0.28	0.06	0.56	0.46	**< 0.01**
			SaO_2_ time < 90%	0.00	0.00	0.09	0.07	0.47
PWA drop time index	0.36	< 0.01*	Body Mass Index	0.06	0.06	0.09	0.09	0.34
			Age (years)	0.05	0.05	0.10	0.09	0.33
			Respiratory Arousal Index	0.23	0.05	0.57	0.46	**< 0.01**
			SaO_2_ time < 90%	0.00	0.00	0.05	0.43	0.74
HRV LF/HF ratio (log)	0.25	< 0.01*	Body Mass Index	0.00	0.00	0.10	0.09	0.40
			Age (years)	0.03	0.00	0.44	0.40	**< 0.01**
			Respiratory Arousal Index	0.01	0.00	0.36	0.31	**< 0.01**
			SaO_2_ time < 90%	0.00	0.00	0.21	0.17	0.17
Morning blood glucose (mmol/L)	0.26	< 0.01*	Body Mass Index	0.02	0.03	0.07	0.07	0.53
			Age (years)	0.01	0.02	0.08	0.07	0.48
			Respiratory Arousal Index	0.02	0.02	0.16	0.13	0.21
			SaO_2_ time < 90%	0.00	0.00	0.34	0.26	**0.01**

*Significant model (p < 0.05) after false discovery rate adjustment; *log* logarithmic transformation, *R*^*2*^ the models' multiple correlations, *B* unstandardised regression coefficient, *β* standardised regression coefficient, *PWA* pulse wave amplitude, *HRV* heart rate variability, *LF* low frequency, *HF* high frequency, *SaO*_*2*_ arterial blood oxygen saturation.

Bolded p-values indicate statistically significant effects

Factors (in Table 3) that were not significantly related to OSA severity or parameters of interest were excluded from further analysis. To identify confounders correlated with the cognitive test results, Pearson’s correlations were calculated (Table 4). These indicated that most of the psychomotor vigilance test (PVT) indices and both of the Austin Maze indices were associated with age. However, for autobiographical memory, age was only associated with episodic memory (total episodic memory, episodic early adult life memory and episodic recent life memory). Smoking was positively correlated with Austin Maze errors and recent life semantic memory. ESS scores were not correlated with performances on any of the cognitive tests.

**Table 4:** Pearson’s correlation analysis between age, smoking, ESS and all cognitive tests.

Variables	Age	Smoking	ESS
PVT RT-mean	R = 0.28 (p = **0.04**)	R = 0.10 (p = 0.38)	R = 0.15 (p = 0.19)
PVT RT slowest 10%	R = 0.19 (p = 0.10)	R = 0.00 (p = 0.94)	R = 0.17 (p = 0.14)
PVT RT-10-lapses > 500 ms	R = 0.26 (p = **0.03**)	R = 0.06 (p = 0.61)	R = 0.19 (p = 0.09)
AM-time (mins)	R = 0.60 (p = ** < 0.01**)	R = 0.14 (p = 0.25)	R = 0.10 (p = 0.39)
AM-errors	R = 0.30 (p = ** < 0.01**)	R = 0.25 (p = **0.02**)	R = 0.10 (p = 0.36)
Total semantic memory	R = − 0.01 (p = 0.91)	R = − 0.17 (p = 0.10)	R = − 0.14 (p = 0.24)
Childhood semantic memory	R = − 0.05 (p = 0.65)	R = − 0.06 (p = 0.15)	R = − 0.00 (p = 0.97)
Early adult life semantic memory	R = 0.05 (p = 0.70)	R = 0.15 (p = 0.20)	R = 0.09 (p = 0.39)
Recent life semantic memory	R = 0.02 (p = 0.86)	R = 0.21 (p = **0.04**)	R = 0.07 (p = 0.50)
Total episodic memory	R = − 0.33 (p = ** < 0.01**)	R = 0.04 (p = 0.70)	R = − 0.02 (p = 0.86)
Episodic childhood memory	R = − 0.19 (p = 0.09)	R = 0.005 (p = 0.96)	R = 0.06 (p = 0.55)
Episodic early adult life memory	R = − 0.25 (p = ** < 0.01**)	R = − 0.04 (p = 0.78)	R = − 0.03 (p = 0.76)
Episodic recent life memory	R = − 0.39 (p = ** < 0.01**)	R = − 0.08 (p = 0.49)	R = − 0.02 (p = 0.81)

*R* correlation, *PVT* psychomotor vigilance test, *RT* reaction time, *AM* Austin Maze, *ms* milliseconds, *ESS* Epworth sleepiness scale.

Bolded p-values indicate statistically significant effects

**Figure 1:**
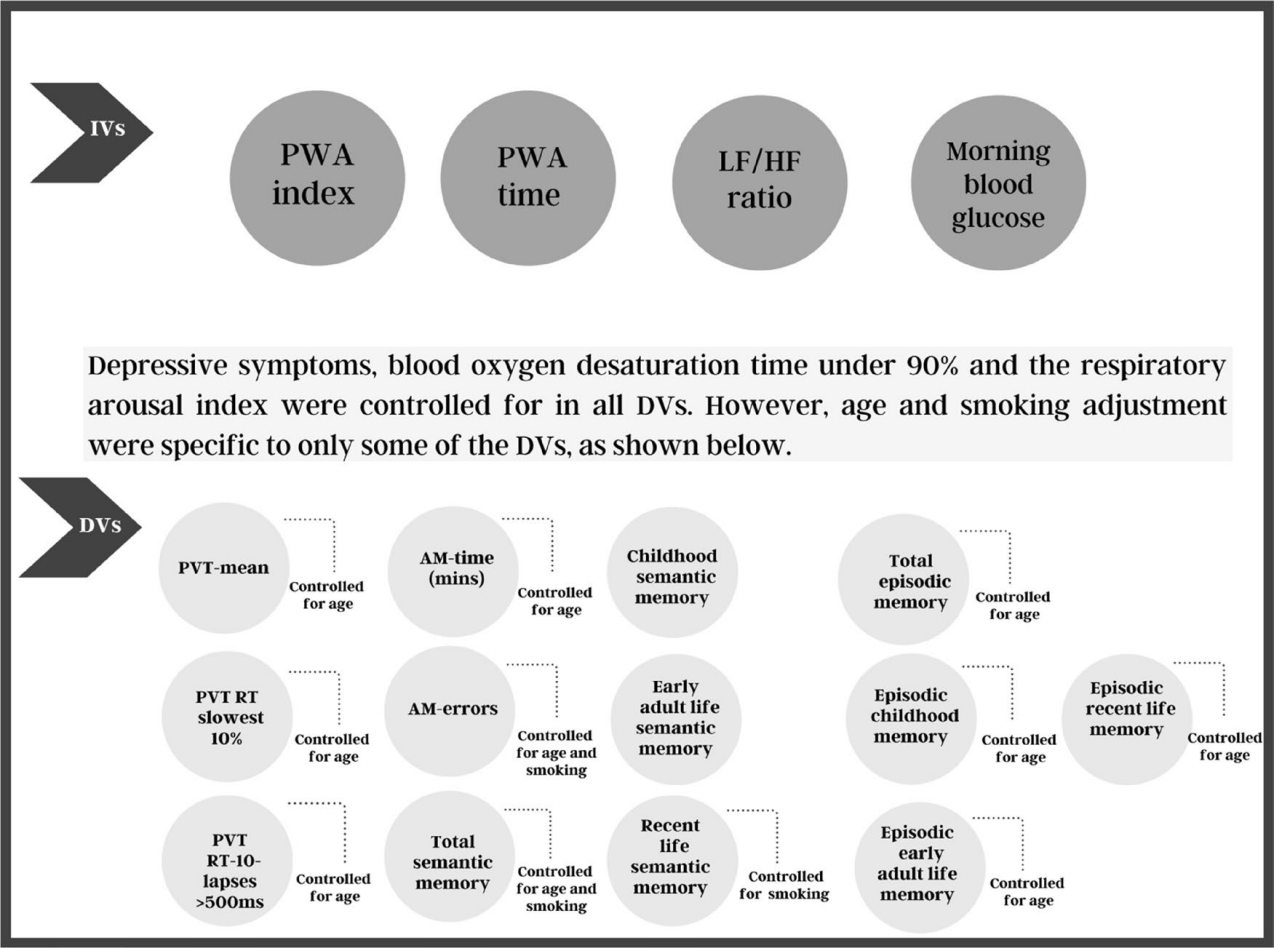
This graph shows the processes of the main analysis for the independent variables, dependent variables and covariates used in the study. *PWA* pulse wave amplitude, *LF* low frequency, *HF* high frequency, *PVT* psychomotor vigilance test, *RT* reaction time, *AM* Austin Maze, *ms* milliseconds, *IVs* independent variables, *DVs* dependent variables.

Multiple linear regression analyses were used to determine the effects of the SNS indices (PWA indices, the HRV LF/HF ratio and morning blood glucose levels) on the PVT, the Austin Maze and autobiographical memory indices after controlling for hypoxia, sleep fragmentation, depression, age and smoking status (Fig. 1; Tables 5 and 6). None of the regression models displayed multicollinearity (variance inflation factors (VIF) < 2.0). After conducting a false discovery rate (FDR) adjustment for multiple comparisons, models that included the PVT, Austin Maze, total semantic memory and recent life episodic memory remained significant (p < 0.05). However, performance on the PVT, autobiographical memory and Austin Maze errors were not associated with any of the nocturnal SNS indices. The Austin Maze time was associated with the PWA index, PWA drop time duration index and the HRV LF/HF (Table 5).

**Table 5:** Multiple linear regression analyses showing the association between the psychomotor vigilance test and Austin Maze and the sympathetic nervous system activity indices.

Cognitive tests	Predictors	R^2^	SE	β	sr	p-value
PVT RT-mean	PWA drops (index)*	0.28	1.11	0.02	0.01	0.87
	PWA drops time durations (index)*	0.28	1.37	0.08	0.06	0.56
	HRV LF/HF ratio (log)*	0.30	8.20	0.08	0.07	0.50
	Morning blood glucose (mmol/L)*	0.27	3.38	0.05	0.04	0.65
PVT slowest 10%	PWA drops (index)*	0.20	4.12	0.11	0.08	0.43
	PWA drops time durations (index)*	0.20	5.07	0.01	0.11	0.92
	HRV LF/HF ratio (log)*	0.22	3.55	0.09	0.08	0.47
	Morning blood glucose (mmol/L)*	0.22	12.30	0.15	0.13	0.22
PVT RT-10-lapses > 500 ms	PWA drops (index)*	0.36	0.12	0.09	0.07	0.47
	PWA drops time durations (index)*	0.36	0.14	0.00	0.00	0.98
	HRV LF/HF ratio (log)*	0.38	0.88	0.14	0.12	0.22
	Morning blood glucose (mmol/L)*	0.36	0.35	0.05	0.04	0.68
AM-10 T-time	PWA drops (index)*	0.45	0.24	0.31	0.29	**< 0.01**
	PWA drops time durations (index)*	0.45	0.39	0.27	0.25	**0.02**
	HRV LF/HF ratio (log)*	0.43	2.04	0.22	0.19	**0.04**
	Morning blood glucose (mmol/L)*	0.45	0.82	0.09	0.07	0.46
AM-10 T-errors	PWA drops (index)*	0.30	0.14	0.07	0.05	0.61
	PWA drops time durations (index)*	0.23	0.18	0.05	0.04	0.72
	HRV LF/HF ratio (log)*	0.25	1.10	0.13	0.12	0.28
	Morning blood glucose (mmol/L)*	0.25	0.44	0.15	0.12	0.25

*Significant model (p < 0.05) after false discovery rate adjustment; The model was adjusted for demographic cofounders, hypoxia, sleep fragmentation and depressive symptoms; *R*^*2*^ the models' multiple correlations, *SE* standard error, *β* standardized regression coefficient, *sr* semi-partial correlation, *log* logarithmic transformation, *PVT* psychomotor vigilance test, *RT* reaction time, *AM* Austin Maze, *ms* milliseconds, *PWA* pulse wave amplitude, *HRV* heart rate variability, *LF* low frequency, *HF* high frequency, *mins* minutes.

Bolded p-values indicate statistically significant effects

**Table 6:** Multiple linear regression analyses of the association between autobiographical memory interview indices and sympathetic nervous system activity indices.

Cognitive tests	Predictors	R^2^	SE	β	sr	p-value
Total semantic memory	PWA drops (index)	0.13	0.09	0.07	0.06	0.62
	PWA drops time durations (index)	0.15	0.11	0.10	0.08	0.47
	HRV LF/HF ratio (log)	0.14	0.67	0.02	0.02	0.86
	Morning blood glucose (mmol/L)	0.14	0.27	0.01	0.01	0.96
Childhood semantic memory	PWA drops (index)	0.15	0.05	0.03	0.02	0.84
	PWA drops time durations (index)	0.16	0.06	0.01	0.00	0.94
	HRV LF/HF ratio (log)	0.20	− 0.35	− 0.22	− 0.21	0.06
	Morning blood glucose (mmol/L)	0.16	0.15	0.02	0.02	0.89
Early adult life semantic memory	PWA drops (index)	0.07	0.05	0.05	0.04	0.75
	PWA drops time durations (index)	0.08	0.06	0.02	0.02	0.88
	HRV LF/HF ratio (log)	0.08	0.37	0.11	0.10	0.38
	Morning blood glucose (mmol/L)	0.08	0.15	0.09	0.09	0.46
Recent life semantic memory	PWA drops (index)	0.12	0.05	0.05	0.04	0.75
	PWA drops time durations (index)	0.12	0.05	0.02	0.01	0.90
	HRV LF/HF ratio (log)	0.12	0.33	0.09	0.08	0.49
	Morning blood glucose (mmol/L)	0.12	0.14	0.06	0.05	0.67
Total episodic memory	PWA drops (index)	0.17	0.12	0.09	0.07	0.53
	PWA drops time durations (index)	0.15	0.15	0.07	0.06	0.60
	HRV LF/HF ratio (log)	0.23	0.87	0.13	0.10	0.09
	Morning blood glucose (mmol/L)	0.15	0.37	0.09	0.08	0.48
Episodic childhood memory	PWA drops (index)	0.02	0.07	0.00	0.00	0.96
	PWA drops time durations (index)	0.01	0.06	0.03	0.02	0.86
	HRV LF/HF ratio (log)	0.05	0.39	0.20	0.19	0.13
	Morning blood glucose (mmol/L)	0.06	3.80	0.12	0.10	0.38
Episodic early adult life memory	PWA drops (index)	0.17	0.05	0.13	0.10	0.36
	PWA drops time durations (index)	0.10	0.06	0.09	0.08	0.50
	HRV LF/HF ratio (log)	0.18	0.36	0.16	0.14	0.14
	Morning blood glucose (mmol/L)	0.11	0.15	0.03	0.02	0.84
Episodic recent life memory	PWA drops (index)*	0.22	0.05	0.11	0.09	0.42
	PWA drops time durations (index)*	0.20	0.06	0.10	0.08	0.46
	HRV LF/HF ratio (log)*	0.21	0.36	0.17	0.15	0.17
	Morning blood glucose (mmol/L)*	0.20	0.14	0.12	0.10	0.34

*Significant model (p < 0.05) after false discovery rate adjustment; The model was adjusted for demographic cofounders, hypoxia, sleep fragmentation and depressive symptoms; *R*^*2*^ the models' multiple correlations, *SE* standard error, *β* standardized regression coefficient, *sr* semi-partial correlation, *log* logarithmic transformation.

## Discussion

The present study estimated nocturnal over-activity of the SNS through a variety of measures including PWA, HRV and stress response biomarkers. Nocturnal over-activity of the SNS, as measured by PWA and HRV, was linked to visuospatial dysfunction but not to sustained attention, response time or autobiographical memory. Morning blood glucose level was not associated with cognitive impairment.

The current results indicate that the stress associated with an SaO_2_ < 90% is sufficient to elevate blood glucose levels for sustained periods of time and remain elevated upon waking. However, despite confirming the relationship between morning blood glucose levels and time spent with an SaO_2_ < 90%, glucose levels were not associated with any cognitive impairments. However, previous studies of patients without OSA have reported a strong relationship between blood glucose levels and memory impairments^23^ and visuospatial dysfunction^24^.

In this study, HRV and PWA were associated with impairments on a test of visuospatial function, which supports the link between nocturnal over-activity of the SNS and visuospatial dysfunction. A systematic review of HRV and cognitive dysfunction in non-OSA patients by Forte et al.^25^ concluded that lower HRV was associated with poor visuospatial implementation. In contrast, Idiaquez et al.^26^ reported that performance on the Trail Making Test-B, which measures visuospatial skills^27^, and nocturnal over-activation of the SNS were not related to one another in OSA patients^26^. The inconsistency between our findings and those of Idiaquez and colleagues may be attributed to their recruitment of a male-only sample and/or the different measures of the visuospatial function used between their study and ours. Regardless of the reason, our results revealed a robust relationship between SNS over-activation during sleep in OSA patients and performance on the Austin Maze test. This finding suggests that nocturnal over-activity of the SNS should be taken into consideration in future studies of cognitive impairment in OSA patients.

Previous studies of healthy subjects have reported a relationship between sympathetic over-activation and poor performance on memory tests^7,28^ and response times^29^. Our results did not find such associations. This may be because, while other studies examined the effects of acute stress on cognitive performance, we investigated the effect of chronic nocturnal stress on cognitive performance during wakefulness, when stress levels were not elevated. Furthermore, cortisol levels in the blood and urine of our subjects were not associated with any measure of OSA severity or cognitive performance. This indicates that the subjects were not physiologically stressed at the time of waking. Therefore, the relationship between nocturnal over-activity of the SNS and visuospatial dysfunction was likely not the result of acute stress but other factors, such as elevations in nocturnal blood pressure that accompany apnoeic episodes^30^ and are involved in brain injury^31^.

This study has some limitations. First, the non-OSA group was smaller than the OSA group. Although this difference was negated using correlational analyses that treated OSA severity as a continuous variable rather than a categorical one, it is possible that the smaller number of participants without OSA decreased the statistical power. Second, the current study recruited participants who had been referred for a sleep study because they were suspected of having a sleep disorder. This selection bias suggests that our results may not be representative of those from a randomly chosen sample. Third, although the non-OSA group were used in the comparison of polysomnography parameters, they cannot be classified as a healthy control group since they were clinically referred for sleep study. Fourth blood glucose and cortisol measurements were based on samples taken upon waking. It is possible that a more dynamic picture of SNS over-activation could have been obtained by continuously sampling blood throughout the night. Fifth, cognitive tests were performed in the evening (at 5:00 pm) prior to bedtime and may have coincided with the wake maintenance zone for participants, which could have masked cognitive impairment. Sixth, severe sleep deprivation has the potential to affect cognitive function and HRV^32,33^. Since actigraphy or sleep diary data were not collected prior to the PSG study night, some of the variance in cognitive performance and HRV may have been attributable to severe sleep deprivation in some participants. However, we consider this possibility to be unlikely, as the mean ESS score did not differ significantly between the OSA severity groups. Seventh, although the OSA severity was associated with SNS over-activity, medication use was not controlled for. Since only 3 patients were taking medications for asthma or diabetes, this small number is unlikely to have influenced the main outcomes of the study. Finally, the current study did not include brain imaging, so it was not possible to correlate the observed cognitive deficits with structural changes.

## Conclusion

This study examined whether there are associations between nocturnal over-activity of the SNS and cognitive impairments in OSA. Our findings revealed that SNS over-activity during sleep, as measured by PWA and HRV, was linked to visuospatial dysfunction among OSA patients. Morning blood glucose level was not related to impairments on any of the cognitive tests we used. These results suggest that there is an association between SNS activity during sleep and visuospatial dysfunction in OSA. Accordingly, interventions that reduce the nocturnal activation of the SNS may contribute to the improvement of visuospatial functioning in these individuals.

## Methods

### Study participants

A retrospective study was conducted (July to December 2018) of patients suspected to have OSA who had been referred by sleep clinics to the Sleep Medicine and Research Centre, King Abdulaziz University Hospital, Jeddah, Saudi Arabia for overnight polysomnography studies. During this period, consecutive patients were given the opportunity to participate in the study. Potential study participants were excluded if they: (1) used CPAP therapy; (2) had been diagnosed with a neurodegenerative disease; (3) typically slept less than 2 h per night (as the recommended minimum sleep duration for a valid polysomnography is 2 h per night)^34^; (4) were prescribed cardiovascular medications.

Ethical approval for the study was obtained from the Human Research Ethics Committee of the Royal Melbourne Institute of Technology University (ethics reference number: HREC 21,459) and from the King Abdulaziz University Hospital Human Research Ethics Committee (ethics reference number: 395-18). The study was conducted according to the guidelines and regulations stated in the ethics approvals. All participants provided written, informed consent to participate in the study.

A sample size calculation was conducted using G*POWER software 3^35^. A total sample size of 56 subjects was required to test the null hypothesis for a moderate effect size (r = 0.30) and 0.80 power^36^. This minimum sample size was increased to provide extra power for multiple comparisons, and a total of 100 participants were recruited into the study. However, 14 participants did not meet the inclusion criteria, and among the remainder, complete data were obtained for all of the study variables in 74 participants. Medication use was documented for all participants included in the study (Table 7).

**Table 7:** Documented medications that were taken by the patients during the study period and patients’ OSA severity categories.

Patients number (n = 3)	Medications used	Patients’ OSA severity categories
1	Insulin and Diamicron (for diabetes)	Mild
2	Insulin (for diabetes)	Mild
3	Montelukast and Theophylline (for asthma)	Moderate

*OSA* obstructive sleep apnoea.

### Procedures and measurements

Once participants were admitted to the sleep laboratory, height and weight were measured, and subjects completed a series of questionnaires designed to collect demographic information and assess daytime sleepiness and mood levels. Cognitive tests were conducted at 5:00 pm. The time to completion all of the tests ranged from 40 to 45 min for all participants. The cognitive tests were administered in the following order: the PVT, Austin Maze and the autobiographical memory interview. Subjects were prepared for the polysomnography at 10:00 pm. After subjects awoke at 6:00 am, blood and urine samples were taken to measure urinary and blood cortisol levels and fasting blood glucose levels. The same procedures and time of all tests and evaluations in the sleep laboratory were applied to the home studies. For home studies, a certified nurse went to the patient’s home at 6:00 am to take blood and urine samples. The samples were transported to the laboratory in less than 2 h and stored at 4–8 °C during transit.

### Questionnaires

#### Epworth sleepiness scale (Arabic version)^37,38^

The ESS was used to assess the general level of daytime sleepiness. The questionnaire contained eight items, each rated from 0 to 3. Higher numbers represented higher levels of sleepiness, and the eight items were summed to give an overall score. Scores between 0–10 indicated no sleepiness; 11–14 indicated mild sleepiness; 15–17 indicated moderate sleepiness; and scores above 17 suggested severe daytime sleepiness.

### Depression, anxiety, stress scale-21 (Arabic version)^39,40^

The Depression, Anxiety, Stress Scale-21 (DASS) was used to assess the level of three emotional states: depression, anxiety and stress. There is convergent validity between the DASS depression and anxiety subscales and the Beck depression and anxiety inventories^41^.

### Polysomnography evaluation

Overnight polysomnography (SOMNO Medics Plus, SOMNOmedics, Randersacker, Germany) was used to evaluate sleep duration and quality, breathing and other sleep-related parameters. Most participants underwent studies in the sleep laboratory, but five subjects underwent home sleep studies. All participants (including home studies) used the same devices and procedures as subjects in the sleep laboratory and were allowed to conduct the study at home for patient convenience and mobility. For all of the sleep parameters, a sleep technician applied sensors 30 min before sleep time. The polysomnography consisted of continuous recordings from surface leads for electroencephalography (EEG), electrooculography, electromyography (from muscles in the sub-mental space and the tibialis anterior muscles bilaterally) and electrocardiography (ECG). The polysomnography consisted of a 10-channel recording montage (Fp1, Fp2, F3, F4, C3, C4, P3, P4, O1 and O2), which was used to measure EEG activity and left/right electrooculography. The nasal pressure was recorded, and nasal and oral airflow were measured by thermocouple device, chest and abdominal impedance belts measured respiratory muscle effort, a pulse oximeter assessed SaO_2_ and pulse waves, a tracheal microphone measured snoring, and body position sensors measured the sleep position. Polysomnographic procedures were repeated for three participants (using the procedures outlined above) due to technical issues and loss of data during the initial studies.

### Autonomic nervous system measurements

Autonomic nervous system activity was estimated using HRV, PWA, and cortisol and glucose levels, not directly measured at the neuronal level.

#### Heart rate variability

HRV is derived from an analysis of the intervals between regular heartbeats (i.e. the time spent between R-waves—the RR intervals)^42^ to measure autonomic nervous system activity^43^. The spectrum of RR intervals was analysed within two bands: LF at 0.04–0.15 Hz and HF at 0.15–0.4 Hz. Previous studies have indicated that HRV HF power primarily indicates parasympathetic nervous system (PNS) activity, while HRV LF power indicates SNS activity^44,45^. However, more recent studies have indicated that HRV LF can be influenced by parasympathetic activity^46,47^. In addition, HRV HF can exhibit unstable results as a measure of PNS activity, which may be attributable to individual differences, the nature of breathing, and sleeping posture^48–51^. Malliani^52^ has suggested that the HRV LF/HF ratio be considered an index of sympatho-vagal balance. Thus, the present study used only the HRV LF/HF ratio to measure SNS over-activity.

#### Pulse wave amplitude

PWA is a signal obtained from finger plethysmography and is directly and positively associated with blood flow^15^. Decreases in PWA can indicate increased sympathetic activation^53,54^. A ≥ 30% drop in the PWA has been recommended as the cut-off value for identifying arousals and respiratory events in OSA patients^16,55^.

#### Biochemical markers

The stress response during sleep was assessed from a morning urine sample (urinary cortisol) and morning serum cortisol and glucose levels. The urine sample was collected using a sanitised kit, and the serum samples were taken from the median cubital vein between 5:00 a.m. and 6:00 a.m. The blood sampling was performed by a nurse, and urine and blood samples were stored for no longer than 2 h between 2 and 8 °C prior to being assayed.

### Neurobehavioural evaluations

#### Psychomotor vigilance test-10 minute version^56^

PVT is a computerised visual test that evaluates the ability to sustain attention and rapidly respond with a button press to cues presented on a digital screen. The reliability and validity of the 10-min version of the test has been confirmed^56^. The test is sensitive to sleep fragmentation and identifies lapses in sustained attention^57^. Three outcome measures were used: (1) mean response time; (2) mean of the slowest 10% response time; and (3) number of lapses with response times > 500 ms. A response time > 100 ms was considered valid. A false start was recorded when the response time was < 100 ms or when it occurred without a stimulus presentation.

#### Austin maze-10 trials^58^

The Austin Maze is a computerised maze that measures visuospatial ability and memory^59,60^. Subjects plot a course through a chequerboard maze by pushing buttons and identifying the correct order through trial and error. Each time the correct button is pushed, a green light is displayed. When an incorrect button is pushed, a red light is displayed and a buzzer sounds. As previous studies have confirmed that 10 trials are enough to accurately assess visuospatial ability and memory^61^, 10 trials were used in this study.

#### The autobiographical memory interview^62^

An autobiographical memory interview was used to assess both episodic and semantic memory. For this study, memories of three time points in each participant’s lifespan were assessed: childhood (before high school); early adulthood (including career, relationships, marriage and children); and recent life (including present and previous hospital or institution stays over the previous 5 years, as well as recent holidays or travel). Scoring was based on published guidelines^62^. For episodic memory, subjects received a score of 3 for full recall that included specifics of time and place, 2 for recall that was personal but general, 1 for an unclear personal memory, and 0 for no answer or a semantic memory. The maximum possible score for each time period was 9, and the maximum total score was 27. For semantic memory, responses were weighted for the level of detail retained (e.g. house number, street name and district). The maximum possible score for each time period was 21 points, and the total maximum was 63. The autobiographical memory interview has achieved high levels of accuracy, reliability and validity^62^ and was translated from English into Arabic. Two Arabic-speaking researchers reviewed the translation and suggested refinements in terms of expression, phrasing and concepts. To confirm the accuracy of the translation from English to Arabic, the Arabic text was translated back into English by an independent bilingual translator with no knowledge of the topic. A comparison of the original and translated interviews revealed no significant differences in content or meaning.

### Analyses

BMI was estimated according to international standards^63^. The polysomnography results were scored manually according to the American Academy of Sleep Medicine (AASM) 2012 scoring protocol^64^, and the classification of abnormal breathing events was based on AASM’s recommendations^64,65^. Apnoea was defined as ≥ 90% reduction in airflow from baseline for ≥ 10 s. Hypopnoea was defined as a discernible reduction in airflow of ≥ 30% of the pre-event baseline for ≥ 10 s using nasal pressure, as well as an associated reduction in oxygen saturation of at least 3% and/or subsequent arousal. The severity of OSA was estimated from the AHI. The degree of hypoxia was based on the cumulative time (in seconds) spent with an SaO_2_ below 90%, while the degree of sleep fragmentation was assessed using the respiratory arousal index, which was calculated by dividing the total number of respiratory arousals by the number of hours of sleep. The duration of NREM sleep stages N1, N2 and N3 and the duration of the REM sleep stage were analysed. Three technicians verified all polysomnography scores to ensure the quality of the scoring process. The technicians also randomly selected and scored cases to confirm inter-observer reliability and accuracy. Bolded p-values in Tables 1, 2, 3, 4, and 5 indicate statistically significant effects.

HRV was analysed using LAB CHART-PRO analysis software (AD INSTRUMENTS, Sydney, Australia). During the polysomnography, the ECG was continuously acquired at 1 kHz. The ECG record for the entire night's sleep was divided into 5-min segments for analysis. Noise was manually removed. The remaining normal-to-normal RR intervals were analysed using the fast fourier transform algorithm.

Plethysmography data were extracted at 128 Hz during the polysomnography, and artefacts were removed using LAB CHART-PRO analysis software (AD INSTRUMENTS, Sydney, Australia). The analysis was limited to PWA drops of ≥ 30% for more than 3 s and less than 60 s^66^. Nocturnal SNS activity was estimated via two measures: the PWA drop index during sleep, which was calculated by dividing the total number of PWA drops into the number of sleep hours; and the PWA drop time duration index, calculated by dividing the number of sleep minutes by the cumulative minutes spent within PWA drops.

Biochemical markers, including urine cortisol levels, and serum cortisol and glucose levels were analysed in a single laboratory by the same biochemist. The biochemist was blinded to the OSA severity of the study participants. Cortisol samples were analysed using an ATELLICA IM cortisol analyser (SIEMENS HEALTHCARE GMBH, Erlangen, Germany) using a sample volume of 20 µL. Serum glucose was analysed using the DIMENSION VISTA 500 system (SIEMENS HEALTHCARE GMBH). The sample volume was 1.2 µL. The µg/dL units were converted to mmol/L for easy readability.

Statistical analyses were conducted using SPSS version 26 (IBM Corp., Armonk, NY, US). Continuous data were checked for normality. The following variables (found to be non-normally distributed) were log-transformed prior to statistical analysis: the HRV LF/HF ratio, PVT slowest 10%, Austin Maze time, Austin Maze errors, autobiographical childhood semantic memory, autobiographical adult early life memory and autobiographical recent life memory. Data are expressed as means and SDs for continuous variables and as frequencies and percentages for categorical variables. Multivariate analysis of variance using a Bonferroni post-hoc analysis was used to determine significant differences between groups based on OSA levels and demographic variables, depressive symptoms, daytime sleepiness, nocturnal SNS indices, polysomnography parameters and the cognitive tests. Between-group comparisons of categorical data were made using Pearson’s Chi-square tests.

As part of a pre-analysis step, a multiple linear regression analysis was conducted to uncover the predictors of the SNS indices. The predictors were selected on the basis of the main OSA predictors (hypoxia and arousal) and the main demographic variables (age and BMI). Covariates were selected by two methods: (1) they were based on the results of our recent study^6^, which was conducted on the same sample as the current study. Thus, depressive symptoms, hypoxia and sleep fragmentation factors were controlled; and (2) the sensitivity of the common factors in OSA, such as excessive daytime sleepiness and main demographic data (including age and smoking), to the cognitive tests that were determined using Pearson’s correlational analyses.

Multiple linear regression analysis examined the associations between nocturnal SNS indices and cognitive performance while controlling for confounders. All of the models were corrected for multiple comparisons with the FDR^67^, and multicollinearity was demonstrated using a VIF of > 2.0. Accordingly, statistical significance was reported for models that had p < 0.05 after being adjusted for multiple comparisons and/or for models that showed no multicollinearity, as assumed with a VIF of < 2.0.
